# Genetic analysis of a major international collection of cultivated apple varieties reveals previously unknown historic heteroploid and inbred relationships

**DOI:** 10.1371/journal.pone.0202405

**Published:** 2018-09-12

**Authors:** Matthew Ordidge, Pianpool Kirdwichai, M. Fazil Baksh, Edward P. Venison, J. George Gibbings, Jim M. Dunwell

**Affiliations:** 1 School of Agriculture, Policy and Development, University of Reading, Reading, United Kingdom; 2 School of Mathematical, Physical and Computational Sciences, University of Reading, Reading, United Kingdom; University of California Davis, UNITED STATES

## Abstract

Domesticated apple (*Malus* x *domestica* Borkh.) is a major global crop and the genetic diversity held within the pool of cultivated varieties is important for the development of future cultivars. The aim of this study was to investigate the diversity held within the domesticated form, through the analysis of a major international germplasm collection of cultivated varieties, the UK National Fruit Collection, consisting of over 2,000 selections of named cultivars and seedling varieties. We utilised Diversity Array Technology (DArT) markers to assess the genetic diversity within the collection. Clustering attempts, using the software STRUCTURE revealed that the accessions formed a complex and historically admixed group for which clear clustering was challenging. Comparison of accessions using the Jaccard similarity coefficient allowed us to identify clonal and duplicate material as well as revealing pairs and groups that appeared more closely related than a standard parent-offspring or full-sibling relations. From further investigation, we were able to propose a number of new pedigrees, which revealed that some historically important cultivars were more closely related than previously documented and that some of them were partially inbred. We were also able to elucidate a number of parent-offspring relationships that had resulted in a number of important polyploid cultivars. This included reuniting polyploid cultivars that in some cases dated as far back as the 18^th^ century, with diploid parents that potentially date back as far as the 13^th^ century.

## Introduction

The domesticated apple (*Malus* x *domestica* Borkh.) is one of the world’s most widely cultivated temperate fruit, although the genetic basis of its domestication continues to be a subject of debate [1–12]. The consensus is that a wild progenitor of the cultivated form originated in Central Asia and, as part of the domestication process, was disseminated outward from this region, initially by seed during the late Neolithic or early Bronze Age; subsequent dissemination by grafting of elite seedlings, as well as more recent breeding, has resulted in a distribution of cultivated varieties commensurate with the wide ranging cultivation of the crop.

It is in part due to this distribution that germplasm collections of apples exist. The potential for any given cultivar to remain extant for hundreds of years, and to be disseminated across many countries, created a need for collections of representative accessions to allow: the identification and testing of individual cultivars in different environments; the establishment of reliable sources of propagation material for distribution and, as has been better recognised in recent years, to maintain a range of elite germplasm for use in research and breeding efforts. Associated with the longevity and clonal propagation of cultivars is the emergence of somatic mutants, or ‘sports’. Where variants are identified as improvement on the original cultivar (for example, increased pigment in fruit) these are often valued in commercial production. Distinct clones must be retained to act as a source of the sported type.

Debate has been raised around the effect of modern breeding and selection on the diversity that remains available within the cultivated varieties. Noiton and Alspach [13] highlighted that modern breeding efforts had been largely based around a small selection of ‘founders’ and concluded that this reliance could result in reduced genetic diversity and effective inbreeding in the future. However, similar to other perennial fruit crops, apples share a number of characteristics that are thought to have been selected to prevent the loss of diversity caused by inbreeding (reviewed by Miller and Gross and Gaut et al. [14, 15]); specifically relevant are their long juvenile phase and self-incompatibility. The consequent maintenance of cultivars through vegetative propagation greatly reduces the number of generations in the domestication process (relative to seed propagated annual crops). Consequently, domesticated perennial crops tend to retain high levels of genetic diversity when compared to domesticated annuals; for example, Miller and Gross [14] summarise that approximately 95% of the neutral variation from wild populations is retained in domesticated perennial fruit crops, due to a weaker domestication bottleneck. An interesting potential side effect of the clonal propagation of outbreeding perennials, the accumulation of deleterious recessive mutations in a heterozygous state, was highlighted in grape [16]. The authors proposed that this feature contributes to the inbreeding depression associated with grape breeding. An overall maintenance of diversity was generally observed in the early genetic analysis of apple cultivars [1, 17, 18].

More recently, Cornille et al. [9] found high levels of diversity maintained in a range of domesticated cultivars of apple: no significant differences were found between groups classified as cider or dessert, little differentiation was possible between material of different geographical origin, and an overall weak genetic structure was found, altogether failing to support the theory that a genetic bottleneck might have occurred. The lack of a domestication bottleneck was further supported by recent findings using genome re-sequencing [12, 19] although in the earlier of these, the authors were able to distinguish between “old” and “new world” cultivars, “early” and “late” ripening cultivars and cider and dessert cultivars. Other studies have assessed genetic diversity within specific selections of cultivated apples and a range of conclusions, around the possibility to identify clustering and associate this with either geography or use, have been presented [20–28]. Additionally, a number of these studies attempted to use genetic data to elucidate previously unknown parent-offspring relationships.

We aimed to assess the genetic diversity within a large collection of cultivars and seedling varieties (i.e. those without a cultivar name) of apple. The UK National Fruit Collection (NFC) is a significant genetic resource; within the collection are offspring from modern (formal) breeding, and informal breeding (for example, amateur breeding and the breeding efforts of gardeners in the 19^th^ century), old seedling cultivars and a selection of ornamental as well as cider cultivars. Understanding the relatedness of varieties of domesticated apple will increase our understanding of the genetic diversity available within the gene pool and support the increased use of a wide range of germplasm for breeding cultivars in the future.

Diversity Arrays Technology (DArT) is a microarray hybridization-based genotyping technology and has been used to study genetic diversity in a range of species (summarised by James et al. [29] and Cruz et al. [30]). The technology has previously been demonstrated in apple by Schouten et al. [31], who presented the development of the complexity reduction method and validated their findings by combining DArT markers with other markers mapped within known populations. Due to the increased robustness over SNP based technologies, DArT was highlighted as offering particular value for use across genetically diverse germplasm. We therefore utilised the DArT marker technology to investigate the genetic clustering of accessions in the UK NFC.

We also utilised DArT data to assess the genetic similarity between individual pairs of accessions in the collection. Having established baselines for the typical similarity of clonal material and first-degree relatives, we identified a set of relations that were closer than normal, and we subsequently used existing provenance and genetic data to elucidate a number of important inbred and heteroploid (i.e. relating to parents and offspring of differing ploidy) relationships.

## Results

### Identification of K clusters and allocation of accessions

The number of clusters (K) identified by the largest ΔK value following STRUCTURE analysis was three to four. Comparison of the log probability of the data for cluster numbers ranging from 1–40 however, suggested that a set of 20–30 clusters might be most appropriate to capture the major structure in the data, and it was noted that a smaller peak in ΔK was clearly present at K = 25.

**Fig 1:**
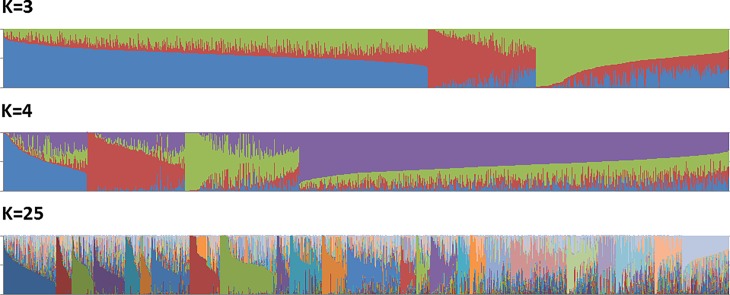
Proportional membership of 2138 accessions within K = 3, K = 4 and K = 25 clusters inferred from STRUCTURE. Each accession is represented by a vertical bar partitioned into K segments representing the proportional membership of the accession within each cluster. Accessions are ordered by cluster of maximal membership and size of maximal proportion.

Two thousand, one hundred and thirty-eight accessions were assigned across three, four and 25 clusters based on their proportion of membership (Fig 1 and S1 Table). A number of patterns were consistent across all clustering alternatives: clonal accessions were clustered together, and a set of major international cultivars and their clones tended to associate with their documented offspring.

Within the three-cluster pattern, clones of ‘Cox’s Orange Pippin’ and its offspring were strongly associated within the smallest (328 members) cluster, clones of ‘Delicious’ and ‘Golden Delicious’ were associated with their respective offspring in the median sized cluster (646 members). Clones of ‘Jonathan’ and ‘McIntosh’ were largely associated with the median sized and largest (1,291 members) clusters respectively, although their proportional association was at approximately 0.7 and 0.6 with the remainder being largely allocated to the smallest and median sized clusters; offspring of these cultivars appeared to follow a similar pattern. Within each cluster were many other accessions and admixture was clear, with only 214 accessions being strongly (≥0.80) associated with any single cluster; 1,777 accessions were at least weakly associated with all three clusters and 100 accessions had a maximal membership within any cluster of below 0.40.

Within the four-cluster pattern, again clones and offspring of ‘Cox’s Orange Pippin’ were associated in a single cluster (the second smallest with 276 members); clones and offspring of ‘Golden Delicious’ and ‘McIntosh’ were associated in the second largest (322 members) and the smallest (230 members) clusters respectively. Clones of ‘Delicious’ were associated at a proportion of approximately 0.5 in each of the smallest and second largest clusters, and clones of ‘Jonathan’ were associated at a proportion of approximately 0.5 within the second largest cluster with the remainder split evenly between the two smallest clusters; again, offspring generally followed this pattern. Significant admixture remained clear and only 194 accessions were strongly (≥0.80) assigned to any single cluster; 926 accessions were, at least weakly, associated with all four clusters and 263 accessions had a maximal membership within any cluster of below 0.40.

Within the 25-cluster pattern, clones of ‘Cox’s Orange Pippin’, ‘Delicious’, ‘Golden Delicious’, ‘Jonathan’ and ‘McIntosh’ were each grouped, along with many of their documented offspring, within different clusters; in each case they were also accompanied by a number of other accessions. Whilst the clones of these major founders were generally strongly associated within their cluster (membership proportion ≥0.98), the remaining members of the cluster generally had assignment proportions of below 0.8. Only 182 accessions were strongly (≥0.80) assigned to any single cluster and 936 accessions had a maximal membership in any cluster of below 0.40. Two hundred and seventy-six accessions were, at least weakly, associated with six or more clusters.

Where both parents were included in the analysis, the offspring were associated with the clusters of each parent, although not always in equal proportion. As an example, ‘Jonagold’ was associated alongside its clones within the cluster containing both ‘Golden Delicious’ and the majority proportional allocation of ‘Jonathan’ (its shared parents) in the three and four-cluster patterns; in the 25-cluster pattern however, the ‘Jonagold’ apportionment was split between the independent clusters containing ‘Golden Delicious’ and ‘Jonathan’ at proportions of approximately 0.64 and 0.35 respectively. Similarly, within all three cluster patterns, a set of ‘Cox’s Orange Pippin’ x ‘Jonathan’ offspring appeared more strongly associated to the cluster containing ‘Cox’s Orange Pippin’ than that containing ‘Jonathan’.

There was no particularly clear association with usage. For example, whilst the documented cider cultivars were generally allocated to a subset of clusters (with ≥91% of them falling within one cluster in the three and four-cluster patterns), their assignment proportion to these was often low and only four (of 109) were assigned at a proportion greater than 0.8. Within the 25-cluster pattern, only 47 cider accessions were assigned to any cluster at a proportion ≥0.40. Furthermore, within each of these clusters were numerous dessert cultivars, although not generally the major founders. The majority of species and/or ornamental accessions (31–42 out of 44) were also assigned to a single cluster in all patterns and 14 of those assigned to a single cluster in the 25-cluster pattern were strongly assigned (≥0.80). Columnar ornamentals also associated with ‘McIntosh’. MM series rootstocks were generally assigned to the same cluster as ‘Northern Spy’.

Some geographic trends were apparent, and the majority of accessions documented as being of American or Canadian origin tended to associate in clusters with ‘Delicious’, ‘McIntosh’ and ‘Jonathan’; 52% of members assigned to the same cluster as ‘Cox’s Orange Pippin’ in the 25-cluster pattern were documented as being of GB origin. Seventeen out of 30 of the accessions documented as originating in Japan had their largest assignment to one of only two clusters in the 25-cluster pattern, although the clusters in question also contained clones of ‘Delicious’ and ‘Jonathan’ respectively. The majority of Japanese accessions also associated in the clusters containing ‘Delicious’ and ‘Jonathan’ in the three and four-cluster patterns.

All provenance, parentage and clonal group membership data are detailed in S1 Table.

### Similarity of accessions by Jaccard coefficient

**Fig 2:**
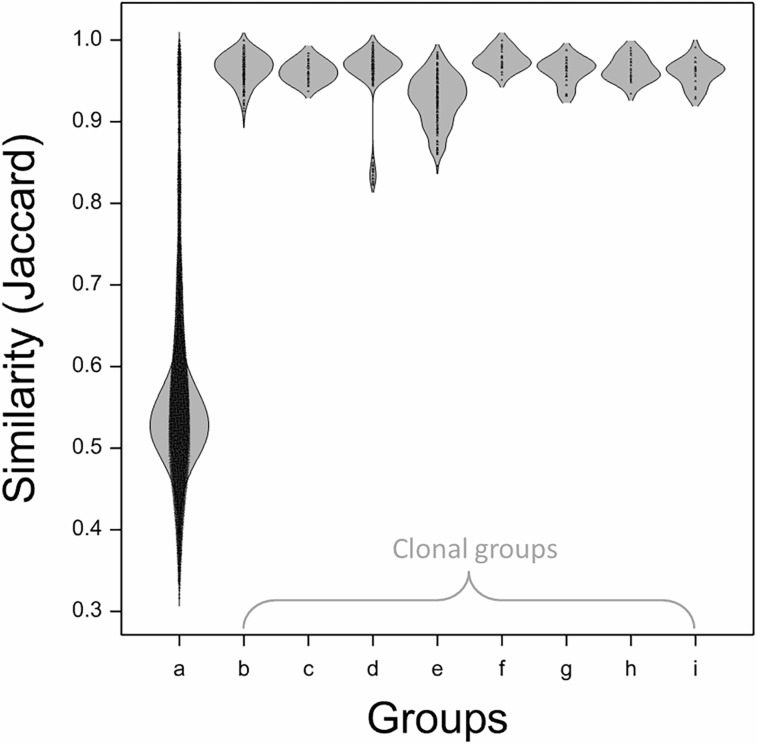
Distribution of similarity across the whole collection (2138 accessions) and a series of documented clones of major international cultivars. 1D Density plot as produced in Genstat using a bandwidth of 0.75. All similarity values were calculated using the Jaccard coefficient. Documented clones were as identified in the NFC database and archive. Groups are as follows: (a) all 2138 accessions including clonal samples; (b-i) clonal samples of ‘Cox’s Orange Pippin’, ‘Delicious’, ‘Golden Delicious’, ‘Jonagold’, ‘Jonathan’, ‘McIntosh’, ‘Northern Spy’ and ‘Rome Beauty’.

Considering similarity between accessions, a score of ≥0.90 was found to generally signify clonality (Table 1). A number of documented ‘Jonagold’ clones scored lower (≥0.85) and one documented clone of ‘Golden Delicious’ (‘Elbee’*) also scored consistently lower (0.82–0.86) against all other clones of ‘Golden Delicious’. The maximum similarity of a non-clonal accession to any of the selected cultivars and clones was 0.90 (for ‘Polly Prosser’ compared to ‘Cox’s Orange Pippin’) but the second highest was 0.87 (and the second highest to any of the ‘Cox’s Orange Pippin’ clones was 0.85). In most cases, the minimum similarity between clones of any cultivar was greater than the maximum similarity of the cultivar against any non-clone. However, the minimum similarity between clones of ‘Jonagold’ was 0.85 and the maximum score between any ‘Jonagold’ clone and any other cultivar was 0.87. The distribution of similarity scores within these clonal subsets was clearly distinct from the majority of the overall population (Fig 2).

**Table 1:** Minimum and maximum similarity of previously documented clones and sports of a set of major cultivars.

Cultivar	No. clones or sports	Maximum clone similarity	Minimum clone similarity	Maximum non-clone similarity
‘Cox’s Orange Pippin’	22	1.000	0.902	0.901
‘Delicious’	10	0.985	0.938	0.790
‘Golden Delicious’	23	0.997	0.823* (0.944)	0.873
‘Jonagold’	20	0.986	0.846	0.866
‘Jonathan’	8	1.000	0.952	0.762
‘McIntosh’	10	0.988	0.932	0.751
‘Northern Spy’	7	0.991	0.935	0.791
‘Rome Beauty’	7	0.991	0.928	0.729

All similarity values were calculated using the Jaccard coefficient. Maximum non-clone values were obtained from a pairwise comparison of all clones to the remainder of the collection.

*The seemingly anomalous value for the ‘Elbee’ clone of ‘Golden Delicious’ is included along with the minimum value from pairwise comparison of the remainder of the documented clones of ‘Golden Delicious’ in parentheses.

#### Identification of further clonal accessions

A series of paired accessions, identified to be indistinguishable by previous SSR comparison [32], were also found to have similarity by DArT analysis of ≥0.90 and these were accepted as either clonal or duplicate; one accession was accepted with a similarity of 0.88 after further morphological comparison. Twenty-six further pairs of accessions scored similarity above 0.90: thirteen of these were deemed to be newly identified duplicates which, on checking, were mostly distinguished only by one or two missing alleles in the SSR data, and six had already been identified as quasi duplicates (generally distinguished by a single allele) in the SSR analysis [32]; five were deemed probable collecting errors in this analysis and these are noted in S2 Table; the remainder was the accession identified as scoring 0.901 in similarity to ‘Cox’s Orange Pippin’ and this was retained for further analysis.

#### Similarity amongst documented offspring and full-siblings

**Fig 3:**
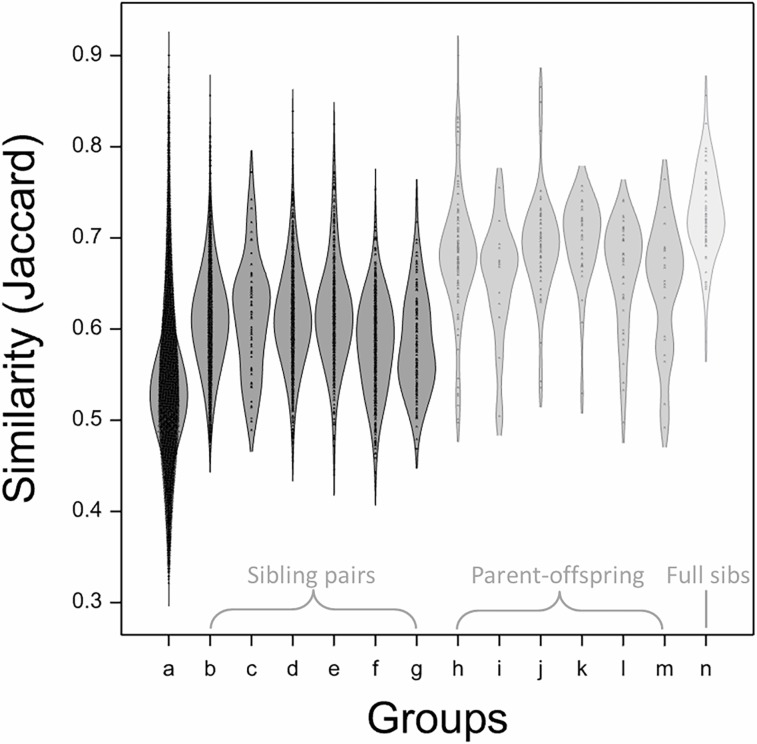
Distribution of similarity across all non clonal or duplicate members of the collection (1777 accessions) and a series of documented siblings, parent-offspring and full-sibs. 1D Density plot as produced in Genstat using a bandwidth of 0.75. All similarity values were calculated using the Jaccard coefficient. Documented relationships were as identified in the NFC database and archive. Groups are as follows: (a) all 1777 accessions including siblings and parents; (b-g) similarity between documented (half- and full-) sibling offspring of ‘Cox’s Orange Pippin’, ‘Delicious’, ‘Golden Delicious’, ‘Jonathan’, ‘McIntosh’ and ‘Worcester Pearmain’; (h-m) similarity between documented offspring of ‘Cox’s Orange Pippin’, ‘Delicious’, ‘Golden Delicious’, ‘Jonathan’, ‘McIntosh’, ‘Worcester Pearmain’ and their respective parent; (n) similarity between documented full-sibling offspring from crosses between ‘Cox’s Orange Pippin’ and ‘Golden Delicious’, ‘Jonathan’ or ‘McIntosh’ and between ‘Jonathan’ and ‘Delicious’ or ‘Golden Delicious’.

First generation offspring shared slightly higher similarity with their siblings than the overall population after removing clones and duplicates (Fig 3), and whilst this was statistically by ANOVA (p<0.001), the difference in mean similarity was not particularly large (0.58–0.62 compared to 0.53). Similarity between documented parents and offspring, and between a set of documented and confirmed full-siblings was sequentially higher (also both significant by ANOVA at p<0.001) with means of 0.65–0.70 and 0.73 respectively. Most notably, this analysis allowed the identification of a subset of relationships scoring higher than that of a standard parent-offspring or full-sibling: 4% (14/332) of these parent-offspring or full-sibling relationships scored similarity ≥0.80 and these were considered further, along with all other relationships scoring between 0.80 and 0.90.

### Accessions with higher similarity than standard parent-offspring or full-siblings

In total, 125 accessions within 47 different groups or pairings were identified to score ≥0.80 but ≤0.90 and these were inferred to have relationships closer than that of a standard parent-offspring or full-sibling but lower than to be accepted as clonal. These could be classified within two groups on the basis of existing ploidy information and/or relationships inferred from documented provenance and existing SSR data:

#### Closely related offspring and siblings

An initial set, mostly consisting of diploid groups and pairs, with one pair of triploids and one mixed group was identified as a set of closely related offspring/siblings (Table 2). From documented pedigrees, four of the groups or pairings were identified as being believed to have parent-offspring or full-sibling relationships and one of the sibling pairs (‘Tydeman’s Late Orange’/‘Merton Pearmain’) was noted to also have a documented inbred relationship (‘Cox’s Orange Pippin’ being the parent of ‘Laxton’s Superb’). Another full-sibling pair (‘Shin Indo’/‘Golden Melon’) were both raised at the same experimental station although there was no documented inbreeding in the known parents. Members of the third full-sibling group were again raised together at the same experimental station and it was notable that the relationships identified as scoring ≥0.80 were of each the three diploid full-siblings to a triploid full-sibling.

**Table 2:** Accessions scoring similarity ≥0.80 and taken to reveal closely related offspring/siblings.

Accession	Cultivar name	Date of origin	Country of origin	Documented parentage	Documented relationship (where known)	Newly proposed relationship	Proposed parentage (newly proposed parents in bold)
**Pairs/groups in agreement with documented relationships**							
1979191	‘Tydeman's Late Orange'	1930	GB	‘Laxton's Superb' x 'Cox's Orange Pippin'	Inbred full-sibling	As documented	As documented
1953029	‘Merton Pearmain'	1934	GB	‘Laxton's Superb' x 'Cox's Orange Pippin'			
1953005	‘Shin Indo' ^(a)^	1930	JP	‘Indo' x 'Golden Delicious'	Full-sibling	As documented	As documented
1953010	‘Golden Melon' ^(a)^	1931	JP	‘Golden Delicious' x 'Indo'			
1971060	‘Karmijn de Sonnaville' ^(^^b^^,^^c^^)^	1949	NL	‘Cox's Orange Pippin' x 'Jonathan'	Full-sibling	As documented	As documented
1974339	‘Leonie de Sonnaville' ^(c)^	pre 1974	NL	‘Cox's Orange Pippin' x 'Jonathan'			
1955004	‘Prinses Marijke' ^(c)^	1935	NL	‘Jonathan' x 'Cox's Orange Pippin'			
1955012	‘Mimi' ^(c)^	1935	NL	‘Jonathan' x 'Cox's Orange Pippin'			
**Pairs/groups with newly proposed relationships **							
1921011	‘Herring's Pippin'	pre 1908	GB	*-*	Parent-offspring	Inbred parent-offspring and inbred half-sibling	**‘Cox's Orange Pippin' x unknown**
1978306	‘Merton Reinette'	1933	GB	‘Cox's Orange Pippin' x 'Herring's Pippin'			‘Cox's Orange Pippin' x 'Herring's Pippin'
1929024	‘Laxton's Pearmain'	1897	GB	‘Cox's Orange Pippin' x 'Wyken Pippin'	Half-sibling	Full-sibling	‘Cox's Orange Pippin' x **'Cellini'**
1982046	‘Ellison's Orange'	pre 1904	GB	‘Cox's Orange Pippin' x 'Calville Blanc'			‘Cox's Orange Pippin' x **'Cellini'**
1945043	‘Laxton's Victory'	1926	GB	‘Cox's Orange Pippin' x 'Exquisite'	Half-sibling	Full-sibling	‘Cox's Orange Pippin' x **'Wealthy'**
1962045	‘Fortune' ^(d)^	1904	GB	‘Cox's Orange Pippin' x 'Wealthy'			‘Cox's Orange Pippin' x **'Wealthy'**
1953057	‘Epicure' ^(d)^	1909	GB	‘Wealthy' x 'Cox's Orange Pippin'			‘Wealthy' x 'Cox's Orange Pippin'
1941006	‘Owen Thomas' ^(e)^	1897	GB	*-*	-	Full-sibling	**‘Cox's Orange Pippin' x 'Gladstone'**
1962020	‘Advance' ^(e)^	1908	GB	‘Cox's Orange Pippin' x 'Gladstone'			‘Cox's Orange Pippin' x 'Gladstone'
1957067	‘James Grieve' ^(d)^	pre 1893	GB	‘Potts Seedling' or 'Cox's Orange Pippin' x unknown	Half-sibling	Inbred parent-offspring / half-sibling	**‘Potts Seedling' x 'Cox's Orange Pippin'**
1979190	‘Sunset'	1918	GB	‘Cox's Orange Pippin' x unknown			‘Cox's Orange Pippin' x '**James Grieve'**
1925031	‘Francis' (Thorrington)	pre 1925	GB	‘Cox's Orange Pippin' x unknown	Inbred half-avuncular	Inbred half-sibling	‘Cox's Orange Pippin' x '**Worcester Pearmain'**
1936037	‘Pearl'	1933	GB	‘Worcester Pearmain' x 'Rival' ^(f)^			‘Worcester Pearmain' x 'Rival'
1974347	‘Grenadier'	pre 1862	GB	*-*	-	Potential sibling	*-*
2000062	‘Lord Grosvenor'	pre 1872	*-*	-			
1949279	‘East Lothian Pippin'	pre 1883	GB	*-*	-	Potential sibling	-
2000088	‘Seaton House'	pre 1860	GB	-			
1958017	‘Lorna Doone' ^(g)^	pre 1958	-	*-*	-	Potential sibling	-
1989076	‘Crimson King' ^(g)^	pre 1895	-	-			

Accession numbers, cultivar names and all provenance data are from the NFC database and archive. Country of origin is abbreviated using the ISO 3166 alpha-2 code.

(a) Both accessions were raised at the Aomori Experimental Station, Japan

(b) accession is a known triploid and the only relationships in this group scoring ≥0.80 were between this and the remaining members

(c) all accessions were raised at the experimental station in Wageningen, Netherlands

(d) accessions analysed were sported forms of the original cultivars but provenance from the original sexually derived cultivar is presented

(e) both accessions were known to have been raised at the same nursery

(f) ‘Cox’s Orange Pippin’ is a documented parent of ‘Rival’

(g) both accessions are triploid. All newly proposed parents are in bold.

Eight further sets, consisting of entirely diploid or triploid pairings or groups, none of which was previously documented to represent closer than a half-sibling relationship, were considered further. A complementary comparison of documented pedigrees and existing SSR data [32] (reproduced in S3 Table) was able to confirm three of the eight as representing plausible full-sibling relationships with incorrectly documented or previously unknown parentage. A further two could be proposed as either a pair of potentially inbred half-siblings or as a possible inbred parent and offspring and the remaining three are proposed as siblings, but since no parental information was available, it was not possible to determine parentage further; the latter of these was a pair of triploid cultivars although there was not clear evidence to suggest a shared diploid gamete.

#### Heteroploid relationships

Thirty-two further groups or pairings with similarity ≥0.80 comprised combinations of diploid and polyploid, or polyploid cultivars. Initially, within these, a set of groupings with documented parentage was recognised (Table 3). These indicated the inheritance of a gamete containing a polyploid (generally diploid) chromosome complement through a parent-offspring relationship. One group of nine accessions was found to have high similarity to ‘Cox’s Orange Pippin’: eight of the members were documented offspring of ‘Cox’ and all were either documented to be triploid or had been identified as triploid from SSR data, with most having also been confirmed by cytometry [32]. Not all pairwise comparisons across the group reported a similarity ≥0.80 but similarity was found to range from 0.70 to 0.86 with the single exception of ‘Polly Prosser’, which was the accession scored at 0.901 in similarity to ‘Cox’s Orange Pippin’ (Table 1).

**Table 3:** Accessions scoring similarity ≥0.80 and taken to confirm documented heteroploid relationships.

Parent			Offspring			Documented parentage of offspring		
Accession	Cultivar name	Ploidy	Accession	Cultivar name	Ploidy			
**Pairs/groups containing a diploid parent**								
2000008	‘Cox's Orange Pippin'	2x	1973297	3022	3x	‘Cox's Orange Pippin'	x	'Northern Spy'
			1960007	‘Carswell's Orange'	3x	″	x	-
			1979163	‘Holstein'	3x	″	x	-
			1972191	‘Jupiter'	3x	″	x	'Starking'
			1971060	‘Karmijn de Sonnaville'	3x	″	x	'Jonathan'
			1974289	‘Oranje de Sonnaville'	3x	″	x	'Jonathan'
			1961058	‘Polly Prosser'	3x	″	x	'Duke of Devonshire'
			1980084	‘Suntan'	3x	″	x	'Court Pendu Plat
1974346	‘Golden Delicious' ^(a)^	2x	1971025	‘Charden'	3x	‘Golden Delicious'	x	'Reinette Clochard'
			2000113	‘Jonagold'	3x	″	x	'Jonathan'
			1977140	‘Mutsu'	3x	″	x	'Indo'
			1974180	‘Sir Prize'	3x	‘Doud Golden Delicious'	x	PRI 14–152
1951250	‘McLiver’s Winesap’^(b)^	2x	1951103	‘(Stayman’s) Winesap’	3x	‘Winesap’	x	-
1953133	‘Ralls Janet'	2x	1953001	‘Fukunishiki'	3x	‘Ralls Janet'	x	'Delicious'
1976149	‘Wagener'	2x	1951044	‘Payette'	3x	‘Ben Davies'	x	'Wagener'
**Pairs/groups containing a triploid parent**								
1973169	‘Belle de Boskoop'	3x	1968057	‘Alfa 68'	4x	‘Belle de Boskoop'	x	'Filippa'
1957215	‘Gravenstein' ^(c)^	3x	1974068	NY E18	4x	‘Red Gravenstein'	x	-
1951103	‘(Stayman’s) Winesap' ^(^^b^^,^^d^^)^	3x	1974070	NY E232	4x	‘Blaxtayman'	x	-
1974341	‘Bramley's Seedling' ^(e)^	3x	1974268	Mather 2	4x	‘Yeoman'	x	-

Accession numbers, cultivar names, ploidy and documented parentage are from the NFC database and archive.

(a) ‘Doud Golden Delicious’ is a known tetraploid form of ‘Golden Delicious’

(b) ‘McLiver’s Winesap’ is deduced to be a sport of ‘Winesap’ and the NFC ‘Winesap’ accession is deduced to be of ‘Stayman’s Winesap’ [discussed below]

(c) the documented parent is a sport of ‘Gravenstein’ but similarity to the original sexually derived cultivar is presented

(d) the documented parent is a sport of ‘Winesap’ but similarity to the original sexually derived cultivar is presented

(e) ‘Bramley’s Seedling’ is a parent of ‘Yeoman’ and therefore a grandparent of ‘Mather 2’.

A second group, identified as having high similarity to ‘Golden Delicious’, contained four members all documented as triploid offspring. Six further pairings with documented parent-offspring relationships were identified; three of these were documented as similar diploid parent-triploid offspring relations and three were of triploid parent-tetraploid offspring. The final pairing with a documented relationship contained ‘Bramley’s Seedling’ and ‘Mather 2’, which is documented as a triploid grandparent-tetraploid grandchild relationship.

In all but the latter of these cases, existing SSR data suggested that cultivars effectively contained a full diploid chromosome complement from the associated parent cultivar, alongside additional alleles presumed to be from the second parent. Where the second parent was also documented this was either able to account for all of the additional (triploid) SSR alleles or was a plausible contributor to a homozygous state for markers where only one or two alleles had been reported. In one of the ‘Golden Delicious’ group, approximately half (5/12) of the SSR markers appeared to have been inherited as per ‘Golden Delicious’ and approximately half (7/12) appeared to have been inherited in a homozygous state, which was consistent with the parent being a named tetraploid sport of ‘Golden Delicious’ (i.e. allowing potential reassortment in the production of the diploid gamete). In this case an SSR profile from the other parent was not available. In the ‘Bramley’s Seedling’-‘Mather 2’ pairing, it appeared that some segregation may have occurred in the intervening generation, and whilst some markers revealed a full matching triploid complement, others only reported two of the grandparent’s triploid alleles. Since the intermediate parent was not available, the reason for this remains unresolved.

Twenty-three heteroploid pairings or groupings without documented relationships were identified (Table 4). Within these, 37 triploid cultivars were associated with 19 potential diploid parents. In all but one of these, existing SSR data (reproduced in S3 Table) supported the plausibility of the inferred relationship and a full diploid parental complement could be found within the triploid profile, although in one case (accession 1947–147) a single allele appeared to be missing. Two pairings suggested the inheritance of a triploid or aneuploid gamete respectively; in the first this was between an accession of ‘Rhode Island Greening’ (a known triploid) and a tetraploid form. In the other (‘Galantine’/‘Serveau’) five out of twelve SSR markers suggested the inheritance of a triploid gamete and four out of twelve were unable to distinguish (because alleles were potentially homozygous) whilst in the remaining three, the third alleles of the proposed parent were apparently missing. Three of the groupings appeared to be triploid siblings that shared a diploid parent, but the parent was not evidently included in the analysis. One exception was found (accession 1949–189), but since this associated within a group of apparent triploid siblings, the accession was documented (and confirmed by cytometry) to be a diploid, and could be excluded as either a potential parent or offspring of any of the group using the existing SSR data, this was assumed to be a sampling error in our analysis and excluded from the group.

**Table 4:** Accessions scoring similarity ≥0.80 and identifying potential polyploid gamete donating parents.

Parent					Offspring					
Accession	Cultivar name	Date of origin	Country of origin	Ploidy	Accession	Cultivar name	Date of origin	Country of origin		Ploidy
2000017	‘Baumann's Reinette’	pre 1811	BE	2x	1996023	‘Joseph Musch’	pre 1872	BE	3x	
1927021	‘Boiken’	pre 1828	DE	2x	1951181	‘Horneburger Pfannkuchen’	-	DE	3x	
1945079	‘Brabant Bellefleur’	pre 1800	BE/NL	2x	1948592	‘Belle-Fleur de France’	-	FR	3x	
					1948595	‘Belle-Fleur Large Mouche’	-	-	3x	
					1983078	‘Dubbele Belle Fleur’	-	-	3x	
					1948312	‘Gros Croquet’	pre 1947	FR	3x	
					1948320	‘Marie Doudou’	-	FR	3x	
					1948286	‘Marroi Rouge’	-	-	3x	
1920020	‘Cornish Aromatic’	pre 1813	GB	2x	1999085	‘Reinette de Brucbrucks’	pre 1947	-	3x	
2000008	‘Cox's Orange Pippin’	1825	GB	2x	1981141	‘Honey Pippin’	pre 1981	GB	3x	
1948328	‘Court Pendu Plat’	pre 1613	EU	2x	1948221	‘Reinette de France’	-	-	3x	
1948656	‘Danziger Kantapfel’	pre 1760	PL	2x	1951056	‘Biesterfelder Renette’	pre 1905	DE	3x	
1953047	‘Dutch Mignonne’	pre 1771	NL	2x	1958058	‘Reinette Coulon’	1856	BE	3x	
1950033	‘Esopus Spitzenburg’	pre 1790	US	2x	1957219	‘King of Tompkins County’	pre 1804	US	3x	
1951167	‘Gelber Munsterlander Borsdorfer’	pre 1951	-	2x	1951168	‘Roter Munsterlander Borsdorfer’	-	-	3x	
2000038	‘Golden Reinette”	pre 1650	EU	2x	1973133	‘Blenheim Orange’	pre 1740	GB	3x	
					1948364	‘Daniel Fele Renet’	-	HU	3x	
					1906033	‘Endsleigh Beauty’	pre 1906	GB	3x	
					1949040	‘Harberts Reinette’	pre 1828	DE	3x	
					1976147	‘Orleans Reinette’	pre 1776	FR	3x	
					1947466	‘Reinette Descardre’	c.1820	BE	3x	
					1924054	‘Beauty of Hants’	pre 1850	GB	3x	
					1958062	‘Kaiser Wilhelm’	pre 1800	DE	3x	
1957218	‘King of the Pippins	pre 1800	FR	2x	2000080	‘Rambour Papeleu’	pre 1853	RU	3x	
					1951055	‘Zabergau Reinette’	1885	DE	3x	
1984011	‘Margil’	pre 1750	EU	2x	1973142	‘Ribston Pippin’	1707	GB/FR	3x	
1920027	‘Nolan Pippin’	pre 1920	-	2x	1970106	‘Ashmead's Kernel’	1700	GB	3x	
					1939028	‘Improved Ashmead's Kernel’ (of Bunyard)	pre 1883	-	3x	
2000072	‘Nonpareil’	pre 1600	FR	2x	1957184	‘King's Acre Pippin’	pre 1897	GB	3x	
1924049	‘Old Pearmain’ (of Kelsey)	pre 1200	GB/FR	2x	1924017	‘Excelsior’	pre 1921	GB	3x	
1947288 / 1948375	Unknown (accessed as ‘Reinette Franche’) / ‘Herceg Batthyanyi alma’	- / pre 1876	- / HU	2x	1948723	‘Carter's Pearmain’	pre 1934	GB	3x	
					1941023	‘Claygate Pearmain’	pre 1821	GB	3x	
					1948234	‘Fremy’	1830	FR	3x	
					1950086	‘Lady Hopetown’	pre 1950	GB	3x	
					1947076	Unknown	-	-	3x	
					1947147	Unknown	-	-	3x	
1948283	‘Verite’	pre 1876	FR	2x	1982287	‘Montmedy’	pre 1864	IT	3x	
1945176	‘Rhode Island Greening’	pre 1650	US	3x	1965044	‘Rhode Island Greening’ (4n)	-	-	4x	
1949153	‘Galantine’	pre 1934	FR	3x	1947203	‘Serveau’	pre 1948	FR	4x	
-	-	-	-	-	1948311	‘Bassard’	pre 1948	FR	3x	
					1948211	‘Pomme de Choux a Nez Creux’	pre 1948	-	3x	
					1948224	‘Pomme de Glace’ (Cher)	pre 1948	-	3x	
-	-	-	-	-	1967054	‘Braddick Nonpareil’	pre 1818	GB	3x	
					1950117	‘Reinette d'Anjou’	pre 1817	BE/DE	3x	
-	-	-	-	-	1947070	‘Osnabrucker Reinette’	pre 1802	DE	3x	
					1944001	‘Wheeler's Russet’	pre 1717	GB	3x	

Accession numbers, cultivar names, provenance and ploidy are from the NFC database and archive. Country of origin is abbreviated using the ISO 3166 alpha-2 code with the exception of EU which is used to signify Europe. Where dates of origin are not documented then the earliest date of record is listed with the prefix ‘pre-‘. Where no date, or country of origin is known to be documented, cells are left empty.

Two additional pairings were identified as scoring similarity ≥0.80 but after further consideration, rescoring of the SSR profiles, and morphological comparison, these were both accepted as pairs of duplicate accessions.

## Discussion

We utilised DArT marker data in two approaches of analysis on the UK’s National Fruit Collection of domestic apples. Initially, a systematic Bayesian clustering approach, using STRUCTURE was used to investigate the collection as a representation of the domesticated apple population. Secondly, we utilised the Jaccard similarity coefficient as a simple measure of genetic similarity, in order to identify a series of specific pairings which were found to reveal a range of inbred and heteroploid relationships that had remained undocumented for many years.

### Number of clusters identified through STRUCTURE analysis

From STRUCTURE analysis, following the approach of Evanno et al. [33] we determined that a group of K = 3 or 4 clusters best represented the distribution of cultivars in the collection. However, we also determined that a grouping of 25 clusters appeared to have some validity. The latter is significantly greater than the number of clusters presented in other recent studies which have tended only to follow the approach of Evanno and identify the maximum ΔK [24–27]. It is notable that in none of these previous studies was the structure revealed by clustering considered to be particularly robust. Additionally, previous studies have often focussed on narrower selections of material. For example, in one case selections were largely restricted to European diploid dessert cultivars in order to retain relevance for breeding dessert apples [26] and others focussed largely on material of French [25], Italian [24] or Danish origin [27]. However, all these studies did also include subsets of wider ranging cultivars as reference. It is possible that our clustering is a reflection of the international nature of the collection and it is worth noting that estimating the number of clusters can be problematic for many datasets; a larger number of clusters can potentially also reveal apparent patterns within the population.

### Clustering by provenance and usage

Numerous studies have explored genetic diversity in smaller sets of domesticated apples. Early studies across groups of 27 and 66 cultivars respectively, reported that genetic clustering generally related to pedigree information [1, 17]. However, the former noted that offspring tended only to be able to be clustered with one of the parents, and the latter also noted that complications were caused by overlapping relationships. Both of these features were observed in our analysis across a substantially wider selection of cultivars.

Oraguzie et al. [18] reported that clustering did not reflect the pedigree or provenance within the genotypes. No clear patterns of clustering were identified in studies by Gaurino et al. [20] or Garkava-Gustavsson et al. [21] whilst clustering was reported to associate with geographic origin by Pereira-Lorenzo et al. [22] and with both parentage and pedigree in a study by Patzak et al. [23]; in the former, the geographic origins in question related to a set of cultivars originating within Spain and the Canary Isles and in the latter, again, cultivars were clustered with only one of the two parental cultivars. In more recent studies using STRUCTURE analysis, Liang et al., [24] found two major groups, each with two subgroups, within a set of diploid Italian germplasm and international reference cultivars. Again, offspring tended to be associated with one parent and, whilst geographic comparisons were not deemed possible, the primary groupings were noted to differentiate between the international reference cultivars and the local Italian germplasm. Lassois et al. [25] reported no clear relationship between geography and genetic structure, although this was largely a study of material within France and the accuracy of historic origins was noted to likely be a confounding factor. Urrestarazu et al. [26] were able to organise diversity into three main groups, which associated with broad geographic regions in a European collection. Subgroupings in this study also appeared to associate with cider production, although it was noted that the major groups themselves were only moderately differentiated. Larsen et al. [27] concluded that there was no genetic structure in a set of Danish local cultivars when compared alongside a series of international references and species accessions, on the basis that only 21% of genotypes were assigned to clusters at a proportion greater than 0.8.

Here, we note some apparent geographical association in clustering, although this appears to be more influenced by association with the major founders than geography per se. As an example, whilst many US and Canadian cultivars associated in clusters with ‘Delicious’, ‘McIntosh’ and ‘Jonathan’ (all of which are of US or Canadian origin), and many GB cultivars associated with ‘Cox’s Orange Pippin’, a series of Japanese cultivars were noted to be assigned to a limited number of clusters containing ‘Delicious’ and ‘Jonathan’. From checking provenance, many of these cultivars were known to be offspring of ‘Jonathan’ and ‘Delicious’ and had been raised through breeding programs at the Aomori Research Station. Their provenance as originating in Japan must be placed within the context of the internationalisation of many of the major founders.

Various studies have attempted to question whether cider cultivars can be distinguished as a grouping. Similar to findings reported by Cornille et al. [9] and Leforestier et al. [34] we found no clear differentiation between cider and dessert cultivars and we would note that the use of apples for cider and dessert has been historically intermixed, at least to some extent. As an example, in the UK, a series of relatively modern cider cultivars have specifically been bred using dessert parents to introduce earliness into selections and whilst none of these were included in our analysis, in an equivalent manner to the ‘MM’ series rootstocks and columnar ornamentals we would expect these to group with their dessert relations. It is likely that equivalents have been produced and/or selected over previous time. Conversely, using a principal components approach, Migicovsky et al. [19] were able to distinguish between cider and dessert cultivars, as well as “old” and “new” world and “early” and “late” cultivars from the USDA collection in Geneva, NY.

### Admixture in clustering

Similar to our findings in apple, Myles et al. [35] previously concluded from analysis of the genetic structure in the UDSA grape germplasm collection, that the domesticated form retained considerable amounts of genetic diversity, with only signs of a weak domestication bottleneck. Diversity in grape cultivars was contained in a complex network of close pedigree relationships and the authors concluded that a large number of first-degree relationships remained in the domesticated gene pool. We detected fewer clonal relationships (Myles et al. [35] detected that 58% of the grape accessions had at least one clonal relative whilst we detected approximately 34% in our sample) and this may represent a slightly more open structure in the germplasm collections of apple. Myles et al. concluded that a significant proportion of the domesticated population represented a single large complex pedigree making the accurate reconstruction of genealogical relationships intractable from genomic data. Whilst there were clear associations between cultivars of known relatedness in our clustering, and noting that where both parents were known, the offspring were often placed in a cluster associated by majority with one parent only, many cultivars of unknown parentage also clustered alongside the sibling offspring of the key founders and it is possible that this reflects a similarly intractable further range of complex pedigrees.

A number of the studies above have attempted to identify parents where SSR data would allow, and Salvi et al. [36] used a combination of SSR data and provenance to reconstruct some significant networks. More recently, Howard et al. [37] used a combination of SNP array data and pedigree data to perform a highly detailed investigation into the pedigree of ‘Honeycrisp’. Our analysis has also allowed us to propose a number of previously undocumented relationships between important historic cultivars and these are discussed further below.

In many of the cited studies, where clustering was allocated by Bayesian analysis, much of the allocation of cultivars to clusters was based on a relatively low proportional membership and admixture was apparent in all cases. Overall, this lack of clear clustering appears to confirm that the selection of elite cultivars, through breeding or otherwise, has likely not resulted in distinct groupings of cultivars based on use, geography or any other characteristic. However, it is important to note that many of the accessions in these studies were not the result of modern breeding. Again, due to the long lived nature of apple cultivars, many old selections exist in collections alongside modern bred and open pollinated seedlings, which may or may not themselves have direct relationships with the major founders. Oraguzie et al., [18] noted that a subset, containing only cultivars, held the same amount of genetic variation as a set containing both cultivars and species accessions, indicating that the cultivated varieties still represented a broader genetic base than predicted by Noiton and Alspach [13]; this finding was also highlighted previously by Dunemann [1] and Hokanson [17].

These findings should not however, detract from the warnings of Noiton and Alspach [13] about the maintenance of diversity in future breeding. It is known that a number of specific cultivars have some degree of inbreeding in their pedigree. Here we identify a number of further potentially inbred cultivars, which scored similarity closer than that of a standard paternal-offspring or full-sibling relation, although the frequency at which we found cultivars scoring this level of similarity remained relatively low (1.5% of non-clonal accessions). Gross et al. [38] noted that no reduction in diversity could be seen from a study of cultivars dating back over the last eight centuries; but also noted that low numbers and diversity of cultivars signified a potential bottleneck that was underway in current commercial production.

Debate continues around the introgression of wild species during both the initial domestication and the subsequent development of cultivated apple varieties [1–12]. In addition to any natural introgression, there has been a significant use of some species types in formal breeding. Examples of this would include the complex pedigrees of cultivars such as ‘Macfree’, ‘Priscilla’, ‘Sir Prize’, ‘Florina’ and ‘Liberty’, which all arose from scab resistance breeding programmes started in the 1940’s [39] and these might be expected to have introgressed genetic material additional to the *Vf* gene from *Malus floribunda*. Interestingly, all of these tended to associate with their other parents including ‘Delicious’, ‘Golden Delicious’, ‘Jonathan’ and ‘McIntosh’ in our clustering. Contrary to this introgression of species diversity into dessert cultivars, ‘Totem’ and ‘Maypole’ are examples of ornamental cultivars that have utilised diversity from within the dessert genepool (specifically the columnar habit of the ‘Wijcik’ mutant of ‘McIntosh’). It is notable that ‘Totem’ and ‘Maypole’ were clustered by majority proportion alongside ‘McIntosh’ in our analysis, with most of their remaining allocation being alongside the majority of the ornamental species.

### Similarity as an indicator of relatedness and clonality

Similarity provided a simple basis on which to identify relatedness in some accessions. In general, a similarity of ≥0.90 was a clear indicator of clonality and this was supported by both provenance and reference to existing SSR data as well as further morphological comparison, the single exception being a documented triploid offspring scoring at the 0.90 borderline. It is notable that a higher level of similarity might be expected for clonal material and the similarity scores obtained from a series of replicate samples taken from one accession of ‘Cox’s Orange Pippin’ and one of ‘Golden Delicious’ scored higher (0.99–1 and 0.97–1 across nine and ten samples respectively).

It is also worth noting that a number of accessions (especially the documented ‘Jonagold’ clones) were accepted as either clonal or duplicate with lower similarity scores of approximately 0.88 and accessions found to have this level of similarity should be considered in detail in further analyses. ‘Jonagold’ was notably the only triploid accession in the set of standards tested and it was considered whether this was a factor specifically associated with triploid clones; similarity between a smaller number of clones of other triploids was compared and was generally higher (0.97 between two clones of ‘Bramley’s Seedling’; 0.92–0.95 between four clones of ‘Blenheim Orange’; 0.91–0.94 between three clones of ‘Belle de Boskoop’ and 0.92–0.94 between three clones of ‘Gravenstein’). It remains unclear as to why the clonal relations of ‘Jonagold’ scored lower. Janick et al. [40] highlight that it is not always known whether the complete genome is present in all triploids and more detailed analysis may allow this to be tested.

Relatedness at the level of parent-offspring or siblings was less straightforward to identify, and although siblings and offspring tended to have higher similarity than the overall population, there was significant potential for overlap. Inferring relatedness based on published pedigrees of old cultivars is also not likely to be error free. In a study on recently bred cultivars and breeding lines, that were generally well documented, Evans et al. [41] found, unsurprisingly that, whilst many of the documented pedigrees were accurate, a number of complications had been caused by complexities in earlier generations. We noted that, of the documented full-siblings initially used for comparison of similarity scores, a number needed to be removed on the basis of existing SSR data. It is likely that equivalent errors remained present in the documented parentage for the group used to compare parent-offspring similarity and this may explain the slight difference in the distribution of parent-offspring and full-sibling scores. It is also worth noting that the verification of relationships by comparison of SSR profiles led, on a number of occasions, to the identification of additional SSR alleles which were previously unscored, especially in accessions that were not known to be triploid at the time of scoring. Nonetheless, that there remained accessions which appeared to score higher similarity than the majority of parent-offspring or full-siblings was clear, and this allowed us to infer a number of previously unknown relationships within some significant historic cultivars.

### Elucidation of incompletely or inaccurately documented relationships

‘Laxton’s Pearmain’ and ‘Ellison’s Orange’ were documented as half-siblings but similarity suggested they are more closely related. From comparison to existing SSR data, both documented paternal parents could be ruled out. Furthermore, on the basis of exclusion, assuming that they were at least full-siblings and that ‘Cox’s Orange Pippin’ was the correctly documented maternal parent, it was possible to identify ‘Cellini’ as the only member of the collection that could have provided all remaining SSR alleles. ‘Cellini’ pre-dates both cultivars, and was well distributed so is therefore a plausible paternal parent of both.

‘Laxton’s Victory’ was found to have high similarity to both ‘Fortune’ and ‘Epicure’, despite being documented as an offspring of ‘Cox’s Orange Pippin’ x ‘Exquisite’ [42]. From further investigation, existing SSR profiles ruled out ‘Exquisite’ as a potential parent, but ‘Wealthy’ remained a plausible paternal parent, suggesting that all three could be full-siblings.

‘Owen Thomas’ and ‘Advance’ were not known to be related but similarity would suggest that they may at least be full-siblings and existing SSR data agreed with the parentage documented for ‘Advance’ in both cases. Similarly, ‘Grenadier’ and ‘Lord Grosvenor’ were not known to be related, but similarity suggested they may at least be full-siblings. In the latter case, neither of the potential parents was able to be identified.

The similarity of ‘James Grieve’ and ‘Sunset’ would suggest a closer relationship than that of a half-sibling and we are able to propose a more complete (and inbred) parentage. Documented records list ‘James Grieve’ as a seedling of either ‘Pott’s Seedling’ [43] or ‘Cox’s Orange Pippin’ (Journal of the Royal Horticultural Society, cited in Smith [42]). We would propose that ‘James Grieve’ is potentially an offspring of both ‘Pott’s Seedling’ and ‘Cox’s Orange Pippin’, and also the previously unknown paternal parent of ‘Sunset’. ‘Cox’s Orange Pippin’ was also identified as a parent of ‘James Grieve’ by Salvi et al. [36]. Recently, Vanderzande et al. [28] found an identity by descent value between ‘James Grieve’ and ‘Kidd’s Orange Red’ that was above their threshold for first degree relations. The authors acknowledged that ‘James Grieve’ and ‘Kidd’s Orange Red’ are documented as half-siblings and that ‘Delicious’ had never been identified as the second parent of ‘James Grieve’. Our findings would appear to exclude the possibility that they are more than half-siblings and our similarity score between them (0.63) does not appear to support a closer relationship. Furthermore, the existing SSR data on our accessions would clearly exclude ‘Delicious’ as a parent of ‘James Grieve’ and on the basis of provenance (fruit of ‘James Grieve’ being first documented in the UK in 1892 [44] and ‘Delicious’ being believed to have arisen in 1880, been introduced in the US in 1895 and arrived in the UK in around 1912 [43]) a parent-offspring relationship would seem implausible.

‘Francis’ and ‘Pearl’ were previously documented to have a half-avuncular relationship but again, similarity suggested that they were closer. ‘Worcester Pearmain’ was a plausible candidate for the unknown paternal parent of ‘Francis’ and this would reveal an inbred half-sibling relationship since ‘Cox’s Orange Pippin’ is also documented as the parent of ‘Rival’ (and thus grandparent of ‘Pearl’).

‘Herring’s Pippin’ and ‘Merton Reinette’ had a documented parent-offspring relationship [45]. Similarity suggested that these were more closely related and existing SSR data confirmed ‘Cox’s Orange Pippin’ as a plausible parent of ‘Herring’s Pippin’ such that an inbred parent/offspring relationship would better explain the similarity score.

In accordance with the above, the similarity between all of the newly proposed parents and their offspring ranged from 0.60 to 0.72 with the exception of ‘Pott’s Seedling’ where no DArT data were available for comparison. It is worth noting that in the existing SSR data for the cultivar ‘Golden Melon’, a single allele (CH02c11) reported a 2 base shift from that of the documented and shared parent ‘Indo’ in our collection. The scoring of this was checked and whilst the data appear to be correct, it was not deemed sufficient to query the documented parentage.

In general, a similarity ≥0.80 was taken to suggest a relationship likely to be closer than that of a standard parent-offspring or full-sibling. Whilst we were able to identify potential inbreeding in some of the groups identified in this study, and were able to identify closer relationships than had been previously documented in others, we did not have sufficient evidence to suggest inbreeding in all of the groups. It is likely that the range of similarity scores associated with inbreeding extends lower, but to avoid false positives, we used this value as a cut-off in our analysis. Nonetheless, that the triploid ‘Karmijn de Sonnaville’ scored ≥0.80 in similarity to all of the three full-sibling diploid members of its group, whilst the similarity between the three diploids (and between all other diploid full-siblings of ‘Jonathan’ and ‘Cox’s Orange Pippin’ tested) was ≤0.80, further suggests that some additional level of relatedness is generally required to produce a similarity ≥0.80. It is possible that the proposed full-sibling relationships presented here may be accentuated through some additional level of historical inbreeding that may be elucidated in more focussed studies of their proposed pedigrees.

### The importance of polyploids in cultivated apple

Many collections of apple cultivars contain a mixture of diploid, triploid and occasionally tetraploid cultivars and in this study we analysed both diploid and polyploid cultivars together. It is notable that numerous polyploid cultivars of apple have survived many years of selection. Janick et al. [40] highlighted that the ratio of triploids amongst commonly grown cultivars is higher than their average rate of occurrence; their inclusion in this study therefore allows a more complete consideration of the diversity in the cultivated type. Amorim et al. [46] analysed genetic similarity between a set of diploid, triploid and tetraploid *Musa* accessions using DArT markers. The markers did not cluster the accessions based on their ploidy groupings and this was also the case in our study. Larsen et al. [27] recently highlighted the challenge in identifying triploid relations from genetic analyses using SSR markers. By including the polyploids in our analysis we were however, able to identify a series of cultivars with heteroploid relationships and some of these have considerable historical importance.

### Elucidation of polyploid relatives

Within the proposed diploid-triploid relations were six cultivars which appeared to be triploid offspring of ‘Brabant Bellefleur’, eight cultivars which appeared to be triploid offspring of ‘Golden Reinette’ and two which appeared to be triploid offspring of ‘King of the Pippins’. Documented dates of origin for the proposed parents ranged from before the 13^th^ century to the modern day and the documented dates of origin for the offspring ranged from the 18^th^ century to the modern day (with some lacking documented dates of origin). Where dates of origin were documented, timelines were generally in agreement with the proposed parentage apart from in one specific case (‘Nolan Pippin’-‘Ashmead’s Kernel’/‘Improved Ashmead’s Kernel’). It is notable that a number of the proposed parents and triploid offspring have considerable history; 19 out of 22 of the proposed parents have provenance in excess of 180 years. Where provenance is available for the polyploid offspring, 25 out of 37 cultivars date back over 100 years, and nine of these date back more than two centuries.

‘Brabant Bellefleur’ is an old and well established cultivar. It was brought to notice at the end of the 18^th^ century [42] and is believed to be of Flemish or Dutch origin. It is well distributed, and it is unsurprising that it might have produced a number of offspring, especially given that the offspring identified here appear to be largely of French origin. ‘King of the Pippins’ is another old and well distributed cultivar and it is also perhaps unsurprising that this has been found as a possible parent of old triploid cultivars. Both Lassois et al. [25] and Urrestarazu et al. [26] also identified ‘King of the Pippins’ as a likely parent of a number of diploid cultivars with previously undocumented parentage.

‘Golden Reinette’ is again an old historic cultivar. Its history is confused [42] but it is believed to have been known in the UK since the 17th century. Again, it is has been well distributed and it is notable that in the NFC the accession of ‘Golden Reinette’ was found to be indistinguishable from an accession of ‘Baxter’s Pearmain’, itself a cultivar stated to date to 1821 [42]. Interestingly, Bultitude [47] noted ‘Baxter’s Pearmain’ as being “in many respects rather like [a] small Blenheim Orange” and Morgan and Richards [45] speculated that ‘Blenheim Orange’ and ‘Orleans Reinette’ could both be offspring of ‘Golden Reinette’ on the basis of shared characteristics. We inferred that the shared parent in this grouping was more likely ‘Golden Reinette’ than ‘Baxter’s Pearmain’ since the provenance of a number of the proposed offspring pre-dates the latter. Either way, it is interesting to find that a number of apples dating back to the 18^th^ century and having arisen from across Europe may all have shared an identifiable parent. We note that Larsen et al. [27] suggest that ‘Orleans Reinette’ could be a parent of ‘Blenheim Orange’, but given the other members of this group, and the observation that they all share the same two SSR alleles as ‘Golden Reinette’, it would seem more plausible on the basis of our study that they are in fact siblings.

An accession of ‘Herceg Batthyanyi Alma’ and another accession, accessed as ‘Reinette Franche’ (although felt not to match published descriptions) were indistinguishable, and were consequently both identified as the potential parent of another group. Given the available provenance, we were unable to resolve the true cultivar name of the parent genotype and this potentially highlights some mislabelling or synonymy in the history of the accessions or cultivars.

‘Nolan Pippin’ appeared to be a potential diploid parent of the two triploid cultivars ‘Ashmead’s Kernel’ and ‘Improved Ashmead’s Kernel’. ‘Nolan Pippin’ however, has no further provenance than having been received by the National Fruit Trials from a Mrs Woodward of Essex, UK in 1920. Its similarity to both ‘Ashmead’s Kernel’ and ‘Improved Ashmead’s Kernel’ (0.84/0.85 respectively) was closer than the similarity between the triploids themselves (0.79). SSR profiles confirmed that ‘Ashmead’s Kernel’ and ‘Improved Ashmead’s Kernel’ are plausible siblings and the diploid allelic complement of ‘Nolan Pippin’ can be found in both. Despite its oldest provenance, it would seem implausible that ‘Nolan Pippin’ and ‘Improved Ashmead’s Kernel’ might have both inherited this matching complement from ‘Ashmead’s Kernel’, especially given ‘Nolan Pippin’s apparent diploid state. Accepting ‘Nolan Pippin’ as a plausible parent would potentially date the cultivar to the 17^th^ century and on that basis, ‘Nolan Pippin’ may not be its original name.

In comparing the similarity of the polyploid relations for which documented parentage was available, it became obvious that the NFC accession of ‘Winesap’ was, in actuality, of ‘Stayman’s Winesap’ (a documented offspring of ‘Winesap’). Both similarity by DArT and SSR identified the NFC ‘(Stayman’s) Winesap’ accession as being clonal to a set of known sports of ‘Stayman’s Winesap’, which is also a known triploid.This fitted with both ‘McLiver’s Winesap’ as being a sport of the original ‘Winesap’ cultivar, and therefore effectively a parent of our ‘(Stayman’s) Winesap’ accession, and our ‘(Stayman’s) Winesap’ accession showing a parental similarity to the seedling NY E232 (Table 3).

In all but one of these cases, the existing SSR data confirmed that the inferred relationship was plausible i.e. that a full diploid complement could be found within the triploid profile, and in the exception (accession 1947–147) only a single allele appeared to be missing. Whilst the existing SSR markers only represent 12 chromosomes, it is interesting to note that all but one polyploid offspring identified therefore, presented a full euploid profile across all SSR markers. Whilst nothing can be determined regarding the gender of parents for those without documentation, it is also noteworthy that in all but one of those with documented parentage, the diploid gamete was supplied by the maternal parent. This is in keeping with the evidence of Chyi and Weeden [48] that triploid hybrids derived from diploid cultivars generally receive the diploid gamete from the maternal parent. Considine et al. [49] found that ova exclusively produced euploid gametes whilst spermatozoa presented both euploidy and aneuploidy. It is possible that the technique we have used is selective in its identification of triploids that contain a full complement and the single example with a missing allele does report another SSR allele, 2bp larger than expected; this could be a result of either aneuploidy or mutation. We also note that the pair of triploids listed in Table 2 (‘Lorna Doone’ and ‘Crimson King’) appear to potentially share a diploid profile for seven out of twelve markers, and this may be worthy of further investigation.

Considine et al. [49] also reported both that the exclusive mode for *Malus* eupolyploidization and aneuploidization is through first-division restitution and that the most likely chromosomes lost in an aneuploid offspring are chromosomes 2, 10 and 17. The missing SSR noted here (CH04e05) is situated on chromosome 7 and if first division restitution is the mechanism behind the polyploidization then it is also possible that the allele could have been lost through a crossing over event. First division restitution acts to retain heterozygosity and the process and value of doing this is reviewed by Bretagnolle and Thompson [50] and Barcaccia et al. [51]. It is possible that further variation will have been introduced into these triploid offspring through a similar mechanism and this would not be expected to be revealed by single SSR alleles. Further genomic comparisons should be able to reveal the extent of the diploid genotype that is retained in the triploid siblings.

In interpreting the potential offspring of triploid parents, some additional queries arose around the formation of the tetraploids ‘Rhode Island Greening’ and ‘Serveau’. Whilst the former appeared as a plausible tetraploid offspring produced from a triploid gamete, no further provenance was available and it is unclear to us how the tetraploid form was originally thought to have arisen. It is notable that the accession arrived to the NFC from Balsgard in 1965 and that much work was done to either select, or force polyploidy in apples in both Sweden and the USA during the 1930-50s. Vaarama [52] discussed the fertilisation of unreduced triploid gametes in the production of artificial tetraploids when considering the origins of the tetraploid ‘Hibernal’. The latter relationship displayed a mixture of SSR alleles, some of which were in agreement with the inheritance of a triploid gamete and others which were not. It is also possible that our proposed relationship is incorrect and that the two cultivars share a diploid parent, and we note that in this case the provenance relating to date of origin is not particularly strong. At this time we are unable to explain these further.

The microarray based DArT data that we used in this analysis provided us with 562 genetic markers. It is clear that the development of sequencing and SNP based genotyping in apple will allow researchers to produce large datasets in the future and, whilst initial approaches have begun to elucidate diploid relatives in fine detail [28, 37] it will be interesting to see whether a more detailed analysis of the heteroploid relationships within domesticated apple reveals further interesting findings.

### Conclusion

Overall, we found that considerable diversity is maintained in the domesticated apple when considering collections of historic cultivars, although in part, this is maintained through a complex mixture of historically admixed relationships. We found that, the simple assessment of genetic similarity by comparing DArT data using a Jaccard coefficient was able to distinguish clonal relationships and was also able to identify a number of relationships that were closer than that of a standard parent-offspring or full-sibling. Some of these appeared to indicate a level of inbreeding and others were a result of the heteroploid relationships that continue to exist between a number of historically important diploid and polyploid cultivars. In doing this we were able to elucidate a number of pedigrees for cultivars that date back as far as the 18th century.

## Methods

### Plant material and DNA extraction

Young leaf material was collected from accessions in the National Fruit Collection, Brogdale Farm, UK. Leaf material was stored at -20°C prior to DNA extraction. DNA extracts were carried out using Macherey Nagel Nucleospin® 96 Plant II kit (Macherey-Nagel GmbH & Co. KG) according to the kit instructions. Samples of purified DNA were supplied to DArT Pty Ltd. for analysis.

### DArT analysis

DNA samples were analysed by DArT Pty Ltd. as a service provision using the DArT cultivated apple array containing 2,688 markers that had been found to be polymorphic within a range of mapping populations and diversity sets of cultivated apple [31]. Samples were submitted and analysed in three batches containing 187, 1,935 and 217 samples respectively. Within these were a number of replicated samples and some repeated samples that had previously failed to report. A total of 2,195 profiles were reported across the three batches and, having removed the replicated and repeated samples (as well as a set of 24 samples which had been incorrectly labelled) a total of 2,138 profiles were retained for analysis. Data were extracted and analysed from scanned microarray images by DArT PTY Ltd. using DArTsoft software and reported to us as binary scores. Over 1,400 markers were reported in at least one of the three batches but data were screened to retain only the markers (959) that reported in all three batches and these were further screened to isolate data for 562 non-redundant markers based on marker sequence comparison information supplied by Henk Schouten (pers. comm).

### Cluster analysis

Data were analysed using STRUCTURE software [53] and R [54]. The parameters for STRUCTURE included a 50,000 burn in period and 10,000 reps. A recessive alleles model with an admixture ancestry model was used. No location information was used in the clustering. Data were treated as dominant markers and irrespective of ploidy. Independent markers were assumed with the marker distribution parameter being estimated by the program. R was used in implementing the method of Evanno et al. [33] and in producing plots from this implementation. No additional R libraries were required. The value of K clusters that captured most of the structure in the data was estimated following both the method of Evanno et al. [33] and by plotting the estimated Ln probability of data against increasing K and selecting the point at which increases in K ceased to lead to meaningful changes in the Ln probability. Accessions were then allocated to clusters based on their proportional membership and they were deemed to be strongly associated within a cluster when proportional membership was ≥0.80. For the purposes of analysing membership, accessions were generally accepted as members at a proportion of ≥0.40 and a proportion of 0.05 was taken as a minimum value for a recognised, but weak association.

### Existing pedigree, provenance SSR and cytometry data

Pedigree and provenance data were largely taken from the National Fruit Collection database (www.nationalfruitcollection.org.uk) and archive and are otherwise cited to pomological references. SSR data and cytometry data were used, again from the National Fruit Collection database and had been produced as reported by Fernandez-Fernandez [32]. The SSR data had been produced using a set of twelve SSR markers, each situated on different chromosomes and had been scored by capillary electrophoresis. Any secondary checks on SSR marker calls were made using newly extracted samples as part of the ongoing curation of the collection following the same protocols, or by re-calling alleles from the original .fsa files in relation to comparable allele calls. An updated SSR dataset as used in the study, and including all re-called alleles is available from the University of Reading research data archive (http://dx.doi.org/10.17864/1947.163).

### Analysis of genetic similarity

Matrices using the Jaccard similarity coefficient were produced in Genstat (VSN International Ltd). An initial matrix was produced including data from all 2,138 accessions (S4 Table). The similarity within each clonal group of eight major international cultivars (‘Cox’s Orange Pippin’, ‘Delicious’, ‘Golden Delicious’, ‘Jonagold’, ‘Jonathan’, ‘McIntosh’, ‘Northern Spy’ and ‘Rome Beauty’), for each of which between seven and 23 known clones or sports were analysed, was compared to establish a baseline acceptance level for determining clonal and/or duplicate accessions. The remainder of the collection, including a number of pairings which had previously been identified as likely duplicates [32], was considered against this acceptance level. Where similarity by DArT suggested that accessions were clearly distinguishable, and where this was supported by morphological comparison, matching SSR profiles were considered to be due to collecting errors and accessions were retained in the analysis. Five pairings were found indistinguishable by DArT but were clearly distinguishable by SSR and morphology, and these were deemed collecting errors in our analysis. Only one from each of these pairs (selected arbitrarily to represent the genotype rather than the accession) was included in further analysis (as indicated in S2 Table). All other accessions deemed to be clonal or duplicate were subsequently removed from further analysis. Similarity in the remaining population (1,777 accessions) was again calculated and within this, the similarity within a set of documented first-generation offspring from a subset of international cultivars (‘Cox’s Orange Pippin’, ‘Delicious’, ‘Golden Delicious’, ‘Jonathan’, ‘McIntosh’ and ‘Worcester Pearmain’) was considered. Relationships which scored ≥0.80 were then identified and each of these relationships was investigated further by analysis of the existing provenance, pedigree and SSR data. An extract of the SSR data used for comparison is reproduced in S3 Table.

## Supporting information

S1 Table Accession details, provenance, parentage and cluster membership proportion across 2138 accessions as included in STRUCTURE analysis for K = 3, 4 and 25 clusters. (XLSX)

S2 Table DArT data scored across the 2138 accessions used in clustering. A yes/no indicator for inclusion in the 1777 member subset is included.
(XLSX)

S3 Table SSR data relating to the newly proposed pedigrees (Tables 2 and 4). Data are reproduced from Fernandez-Fernandez (2010) and the NFC database. Alleles highlighted in grey were adjusted based on recalling or reanalysis during this study.
(XLSX)

S4 Table Matrix of pairwise similarity scores produced for all available accessions (2,138). Similarity scores were calculated using the Jaccard similarity coefficient in Genstat (VSN International Ltd).
(XLSX)
